# Genome-Wide Scanning of Potential Hotspots for Adenosine Methylation: A Potential Path to Neuronal Development

**DOI:** 10.3390/life11111185

**Published:** 2021-11-05

**Authors:** Sanjay Kumar, Lung-Wen Tsai, Pavan Kumar, Rajni Dubey, Deepika Gupta, Anjani Kumar Singh, Vishnu Swarup, Himanshu Narayan Singh

**Affiliations:** 1 Department of Life Sciences, School of Basic Sciences and Research, Sharda University, Greater Noida 201310, India; Sanjay.Kumar7@sharda.ac.in; 2 Department of Medicine Research, Taipei Medical University Hospital, Taipei 11031, Taiwan; lungwen@tmu.edu.tw (L.-W.T.); 205095@h.tmu.edu.tw (R.D.); 3 Department of Information Technology Office, Taipei Medical University Hospital, Taipei 11031, Taiwan; 4 Graduate Institute of Data Science, College of Management, Taipei Medical University, Taipei 11031, Taiwan; 5 Department of Anatomy, All India Institute of Medical Sciences, New Delhi 110029, India; kumarpa@uic.edu; 6 Department of Anatomy & Cell Biology, College of Medicine, University of Illinois, Chicago, IL 60612, USA; 7 Department of Neurology, All India Institute of Medical Sciences, New Delhi 110029, India; deepa12aug@gmail.com; 8 Department of Physics, Atma Ram Sanatan Dharma College, University of Delhi, New Delhi 110021, India; aksingh@arsd.du.ac.in; 9 Department of System Biology, Columbia University Irving Medical Center, New York, NY 10032, USA

**Keywords:** adenosine methylation, m6A, RNA modification, neuronal development

## Abstract

Methylation of adenosines at N6 position (m6A) is the most frequent internal modification in mRNAs of the human genome and attributable to diverse roles in physiological development, and pathophysiological processes. However, studies on the role of m6A in neuronal development are sparse and not well-documented. The m6A detection remains challenging due to its inconsistent pattern and less sensitivity by the current detection techniques. Therefore, we applied a sliding window technique to identify the consensus site (5′-GGACT-3′) *n* ≥ 2 and annotated all m6A hotspots in the human genome. Over 6.78 × 10^7^ hotspots were identified and 96.4% were found to be located in the non-coding regions, suggesting that methylation occurs before splicing. Several genes, *RPS6K, NRP1, NRXN, EGFR, YTHDF2*, have been involved in various stages of neuron development and their functioning. However, the contribution of m6A in these genes needs further validation in the experimental model. Thus, the present study elaborates the location of m6A in the human genome and its function in neuron physiology.

## 1. Introduction

Among the 150 reported RNA modifications to date, methylation at N6 position of adenosine (m6A) is the post-transcriptional RNA modification with a high physiological relevance [1]. This reversible modification of RNA regulates the expression of several genes and affects human physiology [2]. Over 7000 genes have been reported to carry this modification in humans, and aberrant RNA modification contributes to the pathogenesis of various human diseases. Notably, the abnormal modification of human tRNA may lead to mental retardation and intellectual disability [3]. Among all different RNA modifications, m6A modification is most abundant in mRNAs of eukaryotic cells. Altered m6A modifications have been linked with several diseases, such as obesity, cancer, diabetes mellitus, stress-related psychiatric disorders, neuronal development, and functions [4,5]. Several analytical tools have revealed that 5′-GGACU-3′ is the most common structural signature for m6A modification [6,7].

Recent reports demonstrate that not all the adenines in RNA are methylated; the probability of methylation is random, and some RNAs are even entirely devoid of this modification. Moreover, no consensus has been reached for the methylation pattern; nucleotides flanking to “methylable adenines” impact the possibility of their methylation. Cumulatively, these factors cause difficulties in the analysis during in vitro validation of m6A in RNA. In addition, there are several limitations in the current technologies, which are being used for identification of m6A sites. The resolution of methyl-RNA immune-precipitation and sequencing (MeRIP-Seq) covers around 200 nucleotides; therefore, it cannot be used to pinpoint the precise location of the m6A modification [8]. Another technique called site-specific cleavage and radioactive-labeling followed by ligation-assisted extraction and thin-layer chromatography (SCARLET) is time-consuming and expensive and not feasible for high-throughput applications [9,10]. Most existing methods are entirely ineffective in identifying m6A sites due to a biassing and unpredictability of chemicals toward a specific RNA modification, and failure to produce single-nucleotide sequencing data [11,12,13]. Intrinsic features, such as fragility, multiple open reading frames, alternative splicing, and short RNA half-lives contribute to these m6A analysis flaws. Thus, generating all potential m6A sites in a single transcriptome analysis within a predefined time frame is challenging with these currently available tools. Alternatively, tagging the target sequence in the genome itself can unveil the distribution of all potential m6A sites, which display methylation possibilities, and perhaps aiding in the understanding of m6A’s function in physiological processes. Here, we present the sliding window-based technique to identify all adenines in the human genome, considering each one as a potential methylation site. Furthermore, we have also delineated the role of m6A modification in the neurological milieu, contrasting the physiological and pathological conditions.

## 2. Methodology

### 2.1. Definition of m6A Methylation Sites

The consensus sequence (5′-GGACT-3′)n, *n* = 2 in tandem was searched throughout the human genome (version GRCh37 patch 8). If methylated, the two consensus sequences in tandem are considered as more effective in generating physiological effects. Following the strict criteria, no mismatch in the m6A sites was allowed.

### 2.2. PatternRepeatAnnotator: A Home-Made PERL Script

**Figure 1:**
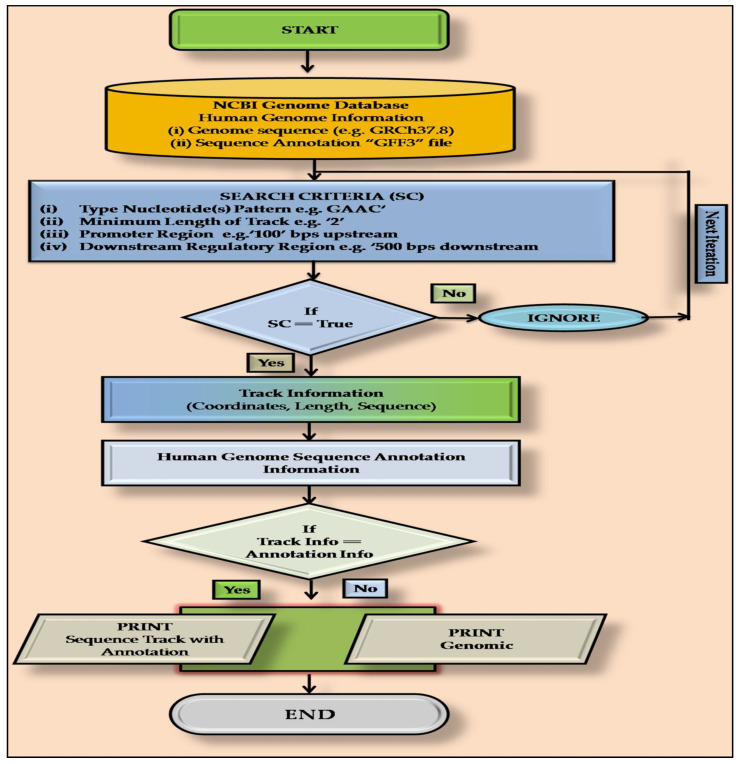
Schematic algorithm used to develop the “PatternRepeatAnnotator”.

To locate m6A sites in the human genome, a home made PERL script, named “PatternRepeatAnnotator” based on the sliding window technique or window shift algorithm was used [14,15]. The ”PatternRepeatAnnotator” was developed to explore the user-defined patterns in the genome sequence (Figure 1). The sliding window technique is a method for finding a subarray (e.g., consensus sequence) in the genome that satisfies the given conditions (e.g., tandem). The search was carried out by maintaining a subset of items (e.g., nucleotides) as a window, and rearranged accordingly and shifted them within the more extensive list until the subarray is precisely matched. The “PatternRepeatAnnotator” scanned the consensus sequences through each chromosome (in Fasta format) to locate them with a particular length (n) defined by the user. Consequently, it provided chromosome-wise coordinates for all the identified sites.

### 2.3. Annotation of m6A Sites

To annotate the identified m6A sites, the GRCh37 genome annotation file was utilized (https://ftp.ncbi.nlm.nih.gov/genomes/archive/old_refseq/Homo_sapiens/ARCHIVE/BUILD.37.3/GFF/ref_GRCh37.p5_top_level.gff3.gz, accessed on 26 September 2021). The identified coordinates of m6A sites were further mapped to the annotation file. After the processing, all information was transported to a comma-separated value (.csv) file, where the running task was conducted. The promoter and downstream regulatory regions (DRR) were considered as 100 nucleotides upstream and 500 nucleotides downstream of all identified genes, respectively. The genes containing recognition sequences in the coding (plus/sense) DNA strand were selected for further analysis only. A single gene was counted as one entry, even if it had the target sequence at multiple locations.

### 2.4. Gene Ontology (GO) Analysis

To assess the mechanistic biological insight into the genes of interest, Gene Ontology (GO) analysis was performed using gprofiler [16]. Enrichment maps were generated using ShinyGo, a gene ontology enrichment analysis software (South Dakota State University, Bioinformatics Research group). The distribution of target sequences (*n* ≥ 2) in protein-coding genes with their frequencies and enrichment score per Mb of respective chromosome were analyzed.

## 3. Results

**Figure 2:**
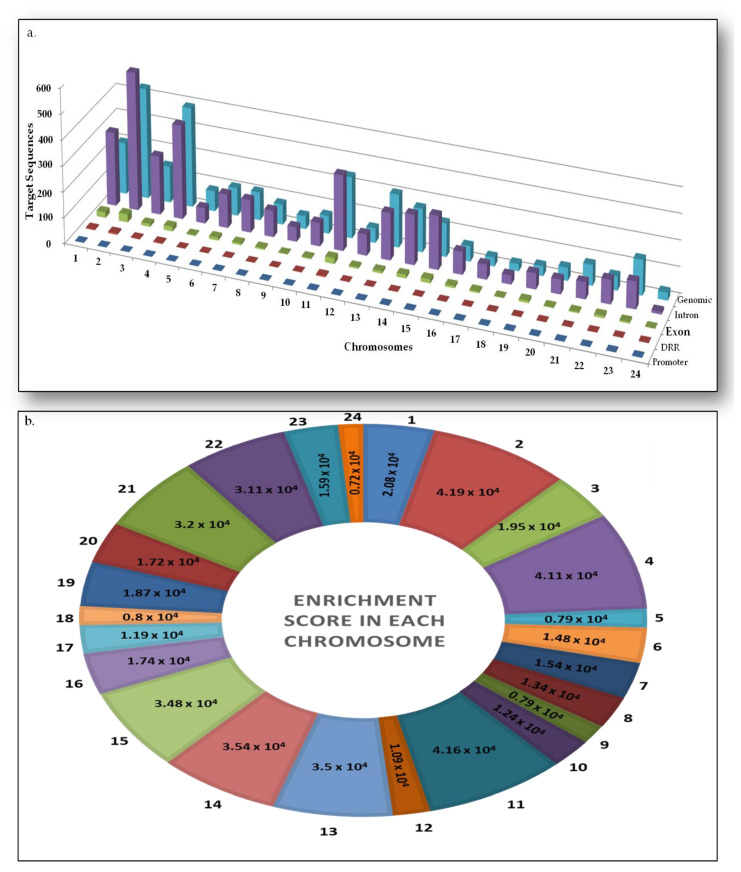
Distribution and enrichment score of m6A sites (**a**). The potential m6A sites (×10^4^) in different parts of human genome, such as promoters, DRR, exons, and genomic (intergenic) regions. (**b**) Enrichment score of target sequences according to chromosome size (in million bases pair). DRR: Downstream regulatory regions.

A total of 6.78 × 10^7^ target sequences GGACT (*n* ≥ 2) were found throughout the human genome using the homemade script. Chromosome 2, having 242 million base pairs (Mbps) nucleotides were found to carry the highest number of target sequences in total (*n* = 1014.79 × 10^4^). Out of these, the target sequences of 31.76 × 10^4^, 541.56 × 10^4^, 1.45 × 10^4^, 433.77 × 10^4^,and 6.23 × 10^4^ Mbps were found in exonic, intronic, promoter, genomic, and downstream regulatory regions (DRR), respectively (Table 1, Figure 2a). The enrichment (copy number of target sequence per Mbps of the chromosome) of target sequence was also found to be highest (4.19 × 10^4^ sequences/Mbps) in chromosome 2 (Figure 2b). Chromosome 24 was found to carry the lowest number of target sequence, in total 41.2 × 10^4^ Mbps with an enrichment score of 0.72 × 10^4^. Out of these, the target sequences 0.07 × 10^4^, 0.31 × 10^4^, 0.67 × 10^4^, 10.31 × 10^4^, and 29.93 × 10^4^ Mbps were identified in promoter, DRR, exonic, intronic, and genomic regions, respectively (Table 1).

Subsequently, we also looked up the protein-coding genes per chromosome, which carry the target sequence (*n* ≥ 2). Here, chromosome 2 had the highest number of genes (*n* = 1448) with the target sequence followed by chromosome 11 (*n* = 982) (Table 2). Interestingly, a notable highest frequency of the target sequence (*n* = 163) was observed in MCF2 Transforming Sequence-Like (*MCF2L*) gene located on chromosome 13. Additionally, the highest number of protein-coding genes were also found on chromosome 13 (81%; 266/327), followed by chromosome 4 (76%; 572/752), whilst chromosome 9 had the lowest number of protein-coding genes with the target sequence (8%; 64/786). Notably, the chromosome 1, containing the highest number of protein-coding genes (*n* = 2058), was found to carry the target sequence only in 27% of genes (Table 2).

Here, the consensus site (5′-GGACT-3′) *n* ≥ 2 was utilized to locate and annotate all m6A hotspots. We identified several genes associated to cancer, diabetes, stress-related mental illnesses, and neuronal development, among other diseases. Especially, GO analysis revealed the crucial genes related to neuronal development.

**Figure 3:**
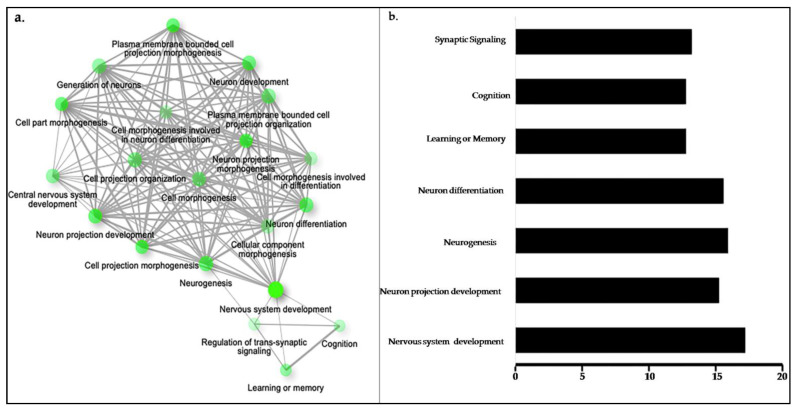
GO analysis of m6A target sites. (**a**) The networking analysis of m6A hotspot genes in different physiological processes. (**b**) Enrichment analysis of m6A hotspot genes for neurological processes, such as neuronal development, neurogenesis, differentiation and projection.

m6A RNA modification is one of the most prevalent reversible internal modifications, regulated by methyltransferases (“writers”) and demethylases (“erasers”) [17]. The presence of complementary seed sequences in micro-RNAs (miRNAs) indicated that miRNAs targeted m6A peak regions in both mouse and human experimental studies.Furthermore, m6A has also been reported in the transcriptome of neurons [9,18]. Brain development is a highly specific and coordinated genetic event andany abnormalities can act as a doorway to different anomalies, such as autistic spectrum and schizophrenia-like disorders [19,20,21]. In our GO analysis data, we selected 1729 genesbased on frequency of target sequence (GGACT) more than 2.Of them, only 27 were scrutinized. The enrichment analysis of the biological process for m6A hotspot genes revealedits association with embryonic brain development, locomotion, neuronal projection, neuronal differentiation, axonal guidance, synaptic assembly, synaptic plasticity, and transmission (Figure 3a,b).

## 4. Discussion

The human genome sequence was explored for all possible m6A sites with two or more target sequences (5′-GGACT-3′) in tandem, which might have a high probability for methylation. The human genome may include some m6A-containing motifs, that still remain unidentified due to their less abundance or beyond the range of advanced detection techniques; hence, surveying the human genome for target sites could be an alternative tool to identify them.

Using the tool “PatternRepeatAnnotator”, a total of 6.78 × 10^7^ target sequences were recognized on the plus strand of the human genome. We observed over representation of the target sequences in non-coding DNA (96.4% in introns, DRR, promoters and genomic regions), whereas a small quantity of 3.5% was located in coding (exonic) regions (Supplementary Figure S1). This internal modification has been reported in nascent pre-mRNAs, suggesting that the addition of methylation group occurs before splicing [22], which is supported by our current findings with 52% target sequences in intronic regions. The m6A modification exhibits spatio-temporal specific expression patterns; therefore, despite many target sequences, only a few undergo methylation [23]. The high density of m6A sites present in 95.8% of intron in non-coding genomic regions, were primarily involved in producing miRNAs. It has been reported that miRNAs influence the fundamental biological processes from cell division to cell death and may undergo m6A modification [24]. For example, m6A modifications in primary miRNA enhance their recognition and processing by DGCR8, a miRNA microprocessor complex protein [25]. Therefore, identified m6A sites may provide deep insight into the mRNA–miRNA interaction pathways involved in the pathogenesis of various diseases. Ribosomal protein S6 kinase genes *RPS6K* have been predicted as a potential candidate for the pathogenesis of hepatocellular carcinoma by the miRNA–mRNA network analysis [26]. This is in line with our enrichment analysis (Supplementary Table S1) identifying RPS6KA3 and RPS6KA5 ribosomal genes, which are associated with regulation of axonogenesis and cellular morphogenesis in the course of neuronal differentiation. Any alteration of m6A methylation of *RPS6KA3* and *RPS6KA5* may affect the normal neurite outgrowth and arborization [27].

Neurexin performs distinct regulatory functions in different classes of neurons, and any mutation or deletion of Neurexin (*NRXN1* and *NRXN2*) genes have been associated with autism-associated behavioral changes in experimental mice [28]. Neurexin also plays a key role in the trafficking of presynaptic vesicles and their deletion resulted in the reduction of synaptic current. To our knowledge, no report exists on the direct link between neurexins and m6A. However, our enrichment analysis data have shown that m6A may regulate *NRXN1*, *NRXN2* and *NRXN3* genes.

In a synaptic epi-transcriptomic study, 4469 enriched m6A sites have been reported selectively in 2921 genes in the forebrain of adult mice and imply that chemically modified mRNA could significantly promote synaptic function [29]. The knockdown of the m6A reader has shown a dramatic change in the spine morphology and dampened the synaptic transmission, there by suggesting its role in synaptic function. Epidermal Growth Factor Receptor (EGFR) belongs to the tyrosine kinase family and is expressed by neuronal and glial cells in different brain regions [30]. During the early development, EGFR is highly expressed in the midbrain and hippocampus, and its increased expression has been also reported in many pathophysiologies, including Alzheimer’s, Huntington’s, Parkinson’s disease, amyotrophic lateral sclerosis, and traumatic brain injury associated with reactive gliosis [31]. Our data have also shown that m6A is enriched with EGFR, which is consistent with previous findings [32]. YT521-B homology domain family 2 (*YTHDF2*) is a m6A reader and directly binds the m6A modification site of EGFR 3′UTR of mRNA and impedes cell proliferation and growth by modulating the downstream ERK/MAPK pathway [32]. The functions of EGFR could also be modulated by other proteins such as *METTL3* and *FTO* [33,34]. Collectively, these data indicated that m6A modification of mRNA is a requisite for the proper physiological functions of EGFR. Further, the MAPK is a key regulator of neurogenesis, which consists of four distinct cascades, ERK1/2, JNK1/2/3, p38, and ERK5. It has been shown that m6A enriched with *MAPK* and *METTL* played a tumour-suppressive role via the p38/ERK pathway. Since, elevated levels of p-38 and pERK in colorectal cancer have displayed the inhibition of cell migration and proliferation after knockdown of METTL [35]. Likewise, *EGFR, YTHDF2* also regulate the MAPK and NF-kB signalling in systemic lupus erythematosus (SLE). YTHDF2 knockdown has been demonstrated to activate MAPK and NF-kB and resulted in a significant increase in pro-inflammatory events in SLE [7,36]. Additionally, the neurological involvement appears in the early stage in SLE, with cognitive impairment being the most prevalent symptom that correlates with disease activity [37].

The identification and quantification of m6A in the transcriptome are tedious, expensive, and associated with many significant systematic errors. To date, well established in vitro methods have encountered several obstacles, including single-nucleotide resolution, a lack of selective chemical reactivities for a specific RNA modification, and lengthy protocols for m6A identification. These challenges are exacerbated by the stability of RNA and the random frequency of methylation. As a result, finding m6A signatures throughout the whole transcriptome is an extremely difficult task. To address these issues, several webtools and algorithms have been developed, which either investigate various databases of m6A sequences or utilize statistical techniques to more precisely locate m6A sites [36,38,39,40,41,42]. Other tools, such as iRNA-AI, iMethyl-PseAAC, iDNA-Methyl, iRNA-Methyl, and iRNA-PseU have been generated also for the identification and annotation of specific sites for adenosine to inosine editing, protein methylation, DNA methylation, N6-methyl adenosine, using pseudo-nucleotide, and RNA pseudouridine, respectively [42,43,44,45]. These tools need a sequence of interest in which the intended modification is sought, and they offer information on whether or not the desired change is feasible in that sequence. The method created in this work scanned the whole human genome for identification of a specific set of nucleotides (target sequence) and generated well-annotated information as output. This tool fundamentally differs in the origin of the hypothesis, concept of algorithm, and the final results compared with all other available techniques.

The Perl-script-based tool “PatternRepeatAnnotator”employed in our study can be customized in several ways: (i) it can be used to search any repeat type (e.g., CAG triplet repeats of Huntington’s disease, GAA repeats of Friedreich’s ataxia, etc.), (ii) the number of such repeats (1 or more) in tandem can be chosen by the user, (iii) range of promoter/downstream regions (in nucleotide length) can be given at user’s choice, (iv) more importantly, the tool is futuristic, and the latest human genome version (>GRCh37 patch 8) can be provided as a template for target sequence search. The results are stored in a specified folder name after the input sequence, where numerous statistical tools can be applied to analyze data easily. The output file contains well-annotated information, such as (i) identified target sequence viz gene ID, (ii) its symbol, (iii) strand (plus/minus), (iv) location in chromosome (exon/intron/genomic/promoter/downstreamregions), (v) the position of repeat (start to end), (vi) its total length (nucleotides long) and (vi) the sequence itself. Using this robust annotated information, the analysis becomes easier, and the genes of interest can be directly picked up from the desired chromosome for further analysis. This, in turn, reduces the cost, time, and manpower required to evaluate the whole transcriptome for m6A modification. The ability to analyze databases in future depicts long-lived applicability, highly customizable interface, making it user-friendly and robust with rich annotated data.

## 5. Conclusions

The m6A is a conservative phenomenon and has been involved in modulating translation efficiency, mRNA turnover, RNA splicing, miRNA and other non-coding RNA biogenesis. As demonstrated in our study, “PatternRepeatAnnotator”could identify and annotate all “methylable adenosines” in the genome, however, their regulation in vivo needs to be verified as not all m6A sites are modified in the human genome. Annotation of these identified m6A sites revealed that over 96% m6A were found in non-coding regions, which corroborates their roles in downstream regulatory processes. Several essential genes in neuronal development harbor extensive m6A sites. More in vivo investigations are required to correlate these identified m6A sites, their modification pattern, and mechanistic approach in cellular processes and various human diseases.

## Figures and Tables

**Table 1:** Distribution of target sequence (*n* ≥ 2) found in different regions of human genome.

Chromosome Number	Number of Target Sequence ×10^4^					
	Promoter	DRR	Exon	Intron	Genomic	Total
1	1.00	4.17	22.08	289.36	202.29	518.90
2	1.46	6.23	31.76	541.57	433.78	1014.80
3	0.51	2.13	11.55	229.46	142.93	386.58
4	0.90	3.92	18.34	368.27	391.23	782.67
5	0.14	0.13	2.95	60.49	79.17	142.89
6	0.63	0.54	11.49	131.76	108.23	252.65
7	0.38	0.33	7.74	127.44	108.97	244.86
8	0.32	0.27	6.31	103.02	79.42	189.34
9	0.11	0.10	2.29	56.21	50.51	109.22
10	0.23	0.20	4.89	91.10	69.49	165.92
11	1.16	4.89	23.13	293.85	238.57	561.61
12	0.27	0.23	5.90	82.65	55.61	144.66
13	0.52	0.45	9.64	183.52	205.59	399.72
14	0.80	0.68	13.88	194.32	168.73	378.41
15	0.71	0.59	15.63	208.53	129.65	355.11
16	0.42	0.32	8.76	88.48	59.10	157.08
17	0.30	0.24	6.60	57.25	34.28	98.67
18	0.10	0.09	2.06	34.53	27.32	64.10
19	0.44	0.37	9.38	61.79	37.57	109.54
20	0.19	0.16	3.66	56.90	50.03	110.93
21	0.24	0.21	4.74	64.69	79.52	149.41
22	0.47	0.39	9.70	93.20	54.50	158.26
23	0.31	0.28	6.19	105.08	135.69	247.54
24	0.07	0.31	0.67	10.31	29.93	41.29
**Total**	11.68	27.23	239.34	3533.78	2972.11	6784.16
**Percentage of Total**	**0.172**	**0.401**	**3.528**	**52.089**	**43.810**	**100.000**

DRR—Downstream Regulatory Regions.

**Table 2:** Distribution of target sequences (*n* ≥ 2) in protein-coding genes with their frequencies and enrichment score per Mb of respective chromosomes.

Chromosome	Chromosome Size (Mb)	Total No. Protein Coding Genes Present	Number of Protein Coding Genes Carrying Target Sequence (%)	Highest Frequency of Target Sequence in Any Gene	# Enrichment Score × 10^4^
1	249	2058	967 (27)	63	2.08
2	242	1309	1448 (67)	58	4.19
3	198	1078	522 (30)	62	1.95
4	190	752	932 (76)	55	4.11
5	182	876	135 (10)	64	0.79
6	171	1048	497 (26)	32	1.48
7	159	989	352 (21)	51	1.54
8	145	677	286 (25)	73	1.30
9	138	786	99 (8)	88	0.79
10	134	733	226 (18)	43	1.24
11	135	1298	982 (42)	73	4.16
12	133	1034	265 (14)	36	1.09
13	114	327	432 (81)	163	3.50
14	107	830	587 (40)	74	3.54
15	102	613	641 (64)	40	3.48
16	90	873	343 (19)	108	1.74
17	83	1197	261 (12)	21	1.19
18	80	270	92 (18)	35	0.80
19	59	1472	361 (13)	12	1.87
20	64	544	169 (20)	69	1.72
21	47	234	212 (56)	47	3.20
22	51	488	39 (44)	34	3.11
23	156	842	238 (17)	80	1.59
24	57	71	42 (24)	14	0.72

# Enrichment score was calculated as copy number of target sequence per Mbps of chromosome.

## Data Availability

Not applicable.

## Supplementary Materials

The following are available online at https://www.mdpi.com/article/10.3390/life11111185/s1, Figure S1: Percentage distribution of target sequences in different regions of human genome. Table S1: Enrichment Analysis of genes for their biological functions.
