# Supplementary material for: Genome-Wide Scanning of Potential Hotspots for Adenosine Methylation: A Potential Path to Neuronal Development
Source: Life (Basel). 2021 Nov 5;11(11):1185. doi: 10.3390/life11111185 (PMC8618456; doi:10.3390/life11111185)
Supplement: Supplementary file 1 [file life-11-01185-s001.zip › life-1416849-supplementary.pdf]

life

Supplementary Material of Genome-Wide Scanning of Potential Hotspots for Adenosine Methylation: A Potential Path to Neuronal Development

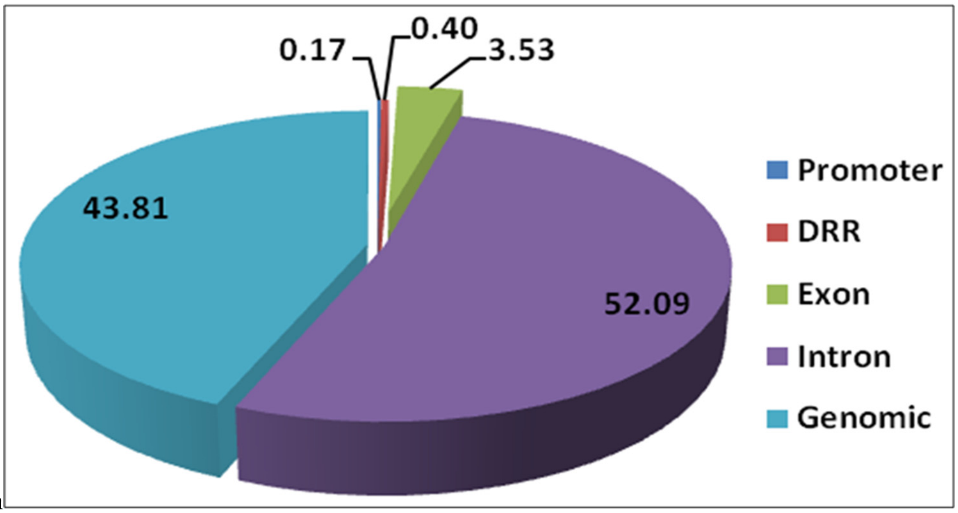

Figure S1. Percentage distribution of target sequences in different regions of human genome.

Table S1. Enrichment Analysis of genes for their biological functions.

Enrichment FDR	Genes in List	Total Genes	Functional Category	Genes
1.98E-20	25	2474	Nervous system development	NTRK3 NLGN4X PAK1 SHANK2 MAP2K1 EP300 NRXN3 CAMK2B PSEN1 DPYSL2 NRP1 MAPK1 SH3GL2 RAP1A NUMB EGFR ADCY1 NRXN1 PLCB1 GRIN2A SIPA1L1 NRXN2 GRIN2B **RPS6KA5 RPS6KA3**
4.63E-18	19	1008	Neuron projection development	NTRK3 PAK1 SHANK2 EP300 PSEN1 DPYSL2 NRP1 SH3GL2 RAP1A NUMB EGFR ADCY1 MAP2K1 SIPA1L1 NRXN1 CAMK2B NRXN3 MAPK1 RPS6KA5
5.91E-18	17	680	Plasma membrane bounded cell projection morphogenesis	PAK1 SHANK2 PSEN1 DPYSL2 NRP1 SH3GL2 NUMB NTRK3 EGFR ADCY1 MAP2K1 SIPA1L1 CAMK2B NRXN3 MAPK1 RPS6KA5 NRXN1
5.91E-18	17	666	Neuron projection morphogenesis	PAK1 SHANK2 PSEN1 DPYSL2 NRP1 SH3GL2 NUMB NTRK3 EGFR ADCY1 MAP2K1 SIPA1L1 CAMK2B NRXN3 MAPK1 RPS6KA5 NRXN1
5.91E-18	17	682	Cell projection morphogenesis	PAK1 SHANK2 PSEN1 DPYSL2 NRP1 SH3GL2 NUMB NTRK3 EGFR ADCY1 MAP2K1 SIPA1L1 CAMK2B NRXN3 MAPK1 **RPS6KA5** NRXN1
7.84E-18	17	701	Cell part morphogenesis	PAK1 SHANK2 PSEN1 DPYSL2 NRP1 SH3GL2 NUMB NTRK3 EGFR ADCY1 MAP2K1 SIPA1L1 CAMK2B NRXN3 MAPK1 **RPS6KA5**NRXN1
1.21E-17	21	1683	Neurogenesis	NTRK3 PAK1 SHANK2 MAP2K1 EP300 PSEN1 DPYSL2 NRP1 MAPK1 SH3GL2 RAP1A NUMB EGFR ADCY1 GRIN2A SIPA1L1 NRXN1 CAMK2B NLGN4X NRXN3 **RPS6KA5**
1.31E-17	20	1412	Neuron differentiation	NTRK3 PAK1 SHANK2 MAP2K1 EP300 PSEN1 DPYSL2 NRP1 SH3GL2 RAP1A NUMB EGFR ADCY1 SIPA1L1 NRXN1 CAMK2B NLGN4X NRXN3 MAPK1 **RPS6KA5**
1.31E-17	19	1154	Neuron development	NTRK3 PAK1 SHANK2 EP300 PSEN1 DPYSL2 NRP1 SH3GL2 RAP1A NUMB EGFR ADCY1 MAP2K1 SIPA1L1 NRXN1 CAMK2B NRXN3 MAPK1 **RPS6KA5**
7.28E-17	20	1552	Plasma membrane bounded cell projection organization	NTRK3 PAK1 SHANK2 EP300 PSEN1 DPYSL2 NRP1 SH3GL2 RAP1A NUMB EGFR ADCY1 MAP2K1 SIPA1L1 NRXN1 CAMK2B GRIN2B NRXN3 MAPK1 **RPS6KA5**
7.28E-17	13	272	Learning or memory	PLCB1 PSEN1 EP300 EGFR SHANK2 ADCY1 **NRXN1** GRIN2A **NRXN3NRXN2** NLGN4X GRIN2B MAPK1
8.24E-17	18	1054	Central nervous system development	SHANK2 PSEN1 DPYSL2 NRP1 MAPK1 NUMB NTRK3 EGFR PAK1 ADCY1 MAP2K1 PLCB1 NLGN4X **NRXN1** GRIN2A GRIN2B SH3GL2 **RPS6KA3**
8.24E-17	20	1575	Generation of neurons	NTRK3 PAK1 SHANK2 MAP2K1 EP300 PSEN1 DPYSL2 NRP1 SH3GL2 RAP1A NUMB EGFR ADCY1 SIPA1L1 **NRXN1** CAMK2B NLGN4X **NRXN3** MAPK1 **RPS6KA5**

8.68E-17	20	1589	Cell projection organization	NTRK3 PAK1 SHANK2 EP300 PSEN1 DPYSL2 NRP1 SH3GL2 RAP1A NUMB EGFR ADCY1 MAP2K1 SIPA1L1 **NRXN1** CAMK2B GRIN2B **NRXN3** MAPK1 **RPS6KA5**
.88E-17	18	1067	Cell morphogenesis	PAK1 SHANK2 PSEN1 DPYSL2 NRP1 EP300 SH3GL2 NUMB NTRK3 EGFR ADCY1 MAP2K1 SIPA1L1 CAMK2B **NRXN3** MAPK1 **RPS6KA5NRXN1**
4.41E-16	18	1172	Cellular component morphogenesis	PAK1 SHANK2 PSEN1 DPYSL2 NRP1 EP300 SH3GL2 NUMB NTRK3 EGFR ADCY1 MAP2K1 SIPA1L1 CAMK2B **NRXN3** MAPK1 **RPS6KA5NRXN1**
4.75E-16	16	756	Cell morphogenesis involved in differentiation	PAK1 SHANK2 PSEN1 DPYSL2 NRP1 EP300 NUMB NTRK3 ADCY1 MAP2K1 SIPA1L1 CAMK2B **NRXN3** MAPK1 RPS6KA5 **NRXN1**
4.75E-16	13	325	Cognition	PLCB1 PSEN1 EP300 EGFR SHANK2 ADCY1 **NRXN1** GRIN2A **NRXN3NRXN2** NLGN4X GRIN2B MAPK1
5.99E-16	15	598	Cell morphogenesis involved in neuron differentiation	PAK1 SHANK2 PSEN1 DPYSL2 NRP1 NUMB NTRK3 ADCY1 MAP2K1 SIPA1L1 CAMK2B **NRXN3** MAPK1 RPS6KA5 **NRXN1**
8.52E-16	14	468	Regulation of trans-synaptic signaling	NLGN4X SHANK2 GRIN2A GRIN2B **NRXN3** PSEN1 MAPK1 RAP1A EGFR ADCY1 **NRXN1** PLCB1 SIPA1L1 CAMK2B
